# Prevalence and correlates of exposure to second hand smoke (SHS) among 14 to 15 year old schoolchildren in a medical officer of health area in Sri Lanka

**DOI:** 10.1186/s12889-018-6148-4

**Published:** 2018-11-07

**Authors:** A. M. A. A. P. Alagiyawanna, Esther Queenie Veerasingam, Nick Townsend

**Affiliations:** 1 Health Promotion Bureau, Ministry of Health, Nutrition and Indigenous Medicine, No 02, Kynsey Road, Colombo, 08 Sri Lanka; 2 Base Hospital, Avissawella, Sri Lanka; 3 Department for Health, University of Bath, Bath, BA2 7AY UK

**Keywords:** Second hand smoking, Passive smoking, Southeast Asia, Sri Lanka, Non-communicable disease, Adolescents, Schoolchildren

## Abstract

**Background:**

Despite reports that Southeast Asia has one of the highest prevalence for childhood exposure to second hand smoke (SHS), there are limited data on SHS exposure among schoolchildren in individual countries in the region, including Sri Lanka. This study aimed to determine the prevalence and correlates of SHS among schoolchildren in a Medical Officer of Health (MOH) region in the country.

**Methods:**

We conducted a cross-sectional study, sampling from nice schools in one MOH region following a two-stage cluster sample design and probability proportionate to size sampling techniques. Data were obtained through a self-completed anonymous questionnaire on socio-demographic and health behaviour risk factors. We achieved an 89.5% response rate, corresponding to a total of 311 students in the final sample.

**Results:**

The prevalence of exposure to SHS during the previous week was 17.6% at home and 25.7% in enclosed public places. There were no significant differences in exposure to SHS between sexes. Univariable analysis found that the presence of smokers at home and mother’s unemployment status were significantly associated with a higher risk of exposure to SHS at home. These variables remained significant in multivariable analysis. Non-Sinhalese ethnicity and presence of smokers at home were significantly associated with exposure to SHS in public places, in both uni- and multivariable analysis. Unemployment status of mother was also found to be a significant determinant of exposure to SHS in public places in multivariable analysis.

**Conclusion:**

Despite numerous antismoking activities and strong antismoking legislation, the prevalence of SHS exposure among schoolchildren is higher in enclosed public places than homes. The implementation and enforcement of antismoking legislation is imperative to tackle this and should be supported by the provision of education for schoolchildren and their families on the health risks of SHS. The high-risk groups identified here could be prioritised for preventive programmes.

**Electronic supplementary material:**

The online version of this article (10.1186/s12889-018-6148-4) contains supplementary material, which is available to authorized users.

## Background

Environmental Tobacco Smoke (ETS), or passive smoking, is commonly attributed to two components: second- and third-hand smoke [1]. Secondhand smoke (SHS) is defined as “the combination of smoke emitted from the burning end of a cigarette or other tobacco products and smoke exhaled by the smoker” [2]. Third hand smoke (THS), however, is a complex phenomenon resulting from residual tobacco smoke pollutants that adhere to the clothing and hair of smokers; to surfaces, furnishings and dust in indoor environments [3].

SHS exposure due to proximity to people smoking, leading to the unintentional inhalation of smoke has major health effects for individuals [4], leading to a population burden in both health and subsequent economic costs when prevalent [5]. It was estimated that 40% of children globally were exposed to SHS in 2004, along with 33% of adult male non-smokers and 35% of adult female non-smokers [6]. Around 600,000 deaths, the equivalent of 1·0% of worldwide mortality, along with 10.9 million disability adjusted life years (DALYs) were attributed to secondhand smoking in that year. Of these secondhand smoking related deaths, 47% occurred in women, 26% in men and 28% in children [6].

The long term effects of smoking on chronic disease are well documented, but the exposure to SHS in adolescents and children can result in more immediate harm through the exacerbation of respiratory conditions such as asthma [7] and respiratory-related school absenteeism [8]. It is imperative, therefore, that we understand both the levels of SHS exposure in this population and the determinants for it, so that appropriate preventative approaches can be developed.

Smoking by household members has been identified as the main predictor for SHS among children [9–14], with family groups and home environments particularly important. Allowing someone to smoke around you [10] is associated with SHS at home, with maternal smoking a stronger predictor than paternal smoking [11, 14, 15]. Low parental education [11, 16] along with the number of cigarettes smoked in the household [11] have also been found to be determining factors. Girls have been found to be at a greater risk of SHS exposure [9, 12], with this more common at older ages [9] and amongst those in lower socioeconomic status [12], for both sexes. Time in the week and year has also been found to make a difference, with a higher prevalence in SHS exposure found on Mondays [11, 12] and during the winter months [11, 12], than at other times. Individual factors, such as self-reported tobacco use status and a lack of awareness about the harmfulness of exposure to second hand smoking from other people [13], have also been found to increase the risk.

The majority of studies on SHS come from high income countries, with few studies collecting information on exposure to SHS among schoolchildren from lower middle-income countries [17–19]. Our understanding of the levels and prevalence of SHS in less affluent countries remains weak, despite this body of evidence. This is a concern as the majority of the global burden attributable to SHS exposure is thought to occur in developing countries in Southeast Asia and the Western Pacific [6].

In 2004 the estimated prevalence of SHS exposure among children in the Southeast Asian region was found to be 53% [6]. However, limited data on SHS exposure among schoolchildren in individual countries within this region remain. The Sri Lanka Global Youth Tobacco Survey (SLGYTS) [18], a national school-based survey of students in grades 8–10 (ages 13 to 15 years), conducted in years 1999, 2003, and 2007, found that exposure to SHS at home and in public places decreased over this time. In 2007, nearly one-third (29.9%) of students reported that they were exposed to SHS in their home during the 7 days prior to the data collection. However, the highest prevalence of home SHS was found in 2003 (51%), increasing from 41% in 1999, so some questions over these large decreases remain. In addition, despite this relatively low prevalence of SHS exposure in 2007, two thirds (66%) of the students reported that they had been exposed to SHS in public places, similar to the prevalence figures from 1999 and 2003 (both 68%). In a separate study, in 2012, Katulanda et al. reported SHS exposure among schoolchildren in grades 10 and 12 (ages 15 to 17 years) in the Colombo district which was lower than found in the SLGYTS at 16.3% [19]. Although this lower prevalence may reflect the impact of The National Authority on Tobacco and Alcohol Act, No. 27, of 2006 [20, 21], differences between the sampled populations of these studies must be recognized. Whereas the SLGYTS sampled from all nine Sri Lankan provinces, to gain a nationally representative sample, the Katulanda et al. study sampled only from schoolchildren in Colombo, the capital city.

The Tobacco and Alcohol Act, No. 27 of 2006, presently practiced in Sri Lanka, has provisions for smoke free environments in indoor public and private places [20, 21]. The main objective of these legal provisions is to protect the public from exposure to SHS. However, the law does not have the provision to ban smoking inside homes and exposure to SHS in homes and indoor public places remains high, despite progress in tobacco control activities [22, 23].

Exposure to SHS amongst adolescents could be a major issue in Sri Lanka due to poor implementation of the anti-smoking legislation and a lack of provision to influence smoking within homes. Substantial health gains could be made by extending effective public health and clinical interventions to reduce SHS in all areas [6]. Previous studies conducted several years ago indicated a reduction of exposure to SHS, but despite many changes in the anti-smoking legislation and approaches to reduce tobacco use, no follow up studies have been carried out. In the present study we aimed to determine the current prevalence of SHS amongst adolescents in one Medical Officer of Health (MOH) region in the country and to examine the factors that determined SHS exposure among government schoolchildren in grades 9 and 10 (ages 14 to 15 years).

## Methods

This study was conducted in the Hanwella MOH region, one of 15 MOH areas in the Colombo district. At the time of data collection Hanwella had a population of 113,807.

The study utilized a two-stage cluster sample design and was conducted among schoolchildren aged 14 (grade 9) to 15 years (grade 10) in government schools. These age groups were included in the study as they can complete the surveys independently and are able to give informed consent. Older students (grades 11 and 13) were excluded as they were preparing for exams (General Certificate of Education (GCE) ordinary level and GCE advanced level tests, respectively) at the time of data collection. In the first stage of sampling, schools were selected proportional to enrollment size. In the second phase, the required number of classes assigned to each school were selected randomly from grade 9 and 10 classes. All students within the selected classes were eligible for participation. The study was conducted between January and March 2016. Private schools were excluded from the study as they make up a low percentage of schools in the country and may have led to sampling bias due to a disproportionate effect on the overall study sample. Eighteen classes were selected from nine schools to obtain the required sample size. Sample size calculations used prevalence data of 16.3% as found in the Katulanda study [24], a 10% margin of error and 95% confidence intervals, as well as accounting for a class level design effect of 1.5 and anticipated non-response of 10%. This resulted in a required sample size of 340 students [25].

We used an anonymous self-completion student survey, which took between 10 and 15 min. The questionnaire was developed for this study (Additional file 1). A pilot was conducted with 21 students prior to the data collection resulting in some questions being removed rephrased or modified according to feedback. Data collection was carried out on the first day of the school week, in order to reduce the chance of absenteeism. Our main outcome variable was self-reported history of exposure to SHS within the 7 days prior to the data collection. Study participants were asked the question: ‘During the last 7 days, did anyone smoke tobacco (cigarette bidi / cigars) inside your home/enclosed public places in your presence?’ [26]. Data were also collected on sex, grade, age, ethnicity, parents’ occupational status and presence of smokers at home.

The collected data were entered into epi-data 3.1 [27] and transferred to SPSS 20.0 [28]. Data analysis was done using SPSS 20.0 software. Descriptive statistics were calculated in the form of frequencies and percentages. A binary logistic regression analysis was performed with exposure to SHS as the dependent variable and individual and socioeconomic factors as independent variables.

## Results

Of the 354 students initially selected, 317 consented to take part in the study. School response rate was 100% and students’ response rate 89.5%. Six questionnaires were excluded from the analysis because of incompleteness (Additional file 2: Figure S1).

Of the 311 remaining respondents, 173 were 14 years old (56.6%), 74.4% were Sinhalese. A significantly higher proportion of female students reported that they had smokers in their household (female = 23%, male = 9%; *p* = 0.001) and had fathers who were unemployed (female = 8%, male = 2%; *p* = 0.01). More than 75% of students in the sample reported that their mothers were employed (Table 1).

**Table 1:** Sample characteristics

Factors	Female n (%)	Male n (%)	Total n (%)	Significance testing
Age				
14 years	90 (56.6)	83 (56.8)	173 (56.6)	χ^2^ = 0.13, df = 1, *p* = 0.72
15 years	69 (43.4)	69 (43.2)	138 (44.4)	
Ethnicity				
Sinhalese	119 (77.8)	108 (71.1)	227 (74.4)	χ^2^ = 1.8, df = 1, *p* = 0.18
Non- Sinhalese	34 (22.2)	44 (28.9)	78 (25.6)	
Presence of smokers in household				
Present	35 (22.6)	13(8.6)	48 (15.7)	χ^2^ = 11.29, df = 1, *p* = 0.001
Not present	120 (77.4)	138 (91.4)	254 (84.7)	
Employment status of father				
Employed	141 (91.6)	144 (98.0)	285 (94.7)	χ^2^ = 6.12, df = 1, *p* = 0.01
Unemployed	13 (8.4)	3 (2.0)	16 (5.3)	
Employment status of the mother				
Employed	112 (72.3)	114 (78.6)	226 (75.5)	χ^2^ = 1.63, df = 1, *p* = 0.20
Unemployed	43 (27.7)	31 (21.4)	74 (24.7)	

Data missing: ethnicity 6, presence of smokers 5, father’s employment status 10, mother’s employment status 11

χ^2^ Chi-square value, *df* degree of freedom, *p* Chi-square test

A higher prevalence of individuals reported to being exposed to SHS in enclosed public places (females = 28%, males = 23%) than at home (females = 20%, males = 15%) although these differences were not found to be significant. Prevalence of exposure to SHS did not differ significantly by age or employment status of father. Even though large differences in the prevalence of SHS exposure were found between those with fathers in employment and not in employment, there were a low percentage of students whose father was unemployed. The presence of smokers at home and unemployed status of the mother were significantly associated with a higher prevalence of exposure to SHS both at home and in public places. Non-Sinhalese ethnicity was significantly associated with a higher prevalence of exposure to SHS in enclosed public places only (Table 2).

**Table 2:** Prevalence of exposure to second hand smoke at home and in public places, Hanwella, Colombo, Sri Lanka

Factors	Home			Public Places		
	Exposed n (%)	Not exposed n (%)	Significance testing	Exposed n (%)	Not exposed n (%)	Significance testing
Total	54 (17.6)	252 (82.4)	P^1^ < 0.0001	80 (25.7)	231 (74.3)	P^1^ < 0.0001
Sex						
Female	31 (20.0)	124 (80.0)	χ^2^ = 1.19, df = 1, *p* = 0.27	45 (28.3)	114 (71.7)	χ^2^ = 1.13, df = 1, *p* = 0.29
Male	23 (15.2)	128 (84.8)		35 (23.0)	117 (77.0)	
Age						
14 years	30 (17.4)	143 (82.7)	χ^2^ = 0.02, df = 1, *p* = 0.87	40 (23.0)	134 (77.0)	χ^2^ = 1.07,df = 1, *p* = 0.30
15 years	24 (18.0)	109 (82.0)		38 (28.1)	97 (71.9)	
Ethnicity						
Sinhalese	42 (18.8)	182 (81.2)	χ^2^ = 1.24, df = 1, *p* = 0.27	52 (22.9)	175 (77.1)	χ^2^ = 5.06, df = 1, *p* = 0.02
Non- Sinhalese	10 (13.2)	66 (86.6)		28 (35.9)	50 (64.1)	
Presence of smokers in household						
Present	24 (50.0)	24 (50.0)	χ^2^ = 41.0, df = 1, *p* < 0.001	20 (41.7)	28 (58.3)	χ^2^ = 8.6, df = 1, *p* = 0.003
Not present	30 (11.6)	228 (88.4)		56 (21.7)	202 (78.3)	
Employment status of father						
Employed	50 (17.9)	230 (82.1)	χ^2^ = 0.3, df = 1, *p* = 0.58	73 (25.6)	212 (74.4)	χ^2^ = 0.25, df = 1, *p* = 0.62
Unemployed	14 (87.4)	2 (12.5)		5 (31.2)	11 (68.8)	
Employment status of mother						
Employed	33 (14.9)	188 (85.1)	χ^2^ = 6.7, df = 1, *p* = 0.01	53 (23.5)	173 (76.5)	χ^2^ = 3.9, df = 1, *p* = 0.05
Unemployed	21 (28.4)	53 (71.6)		26 (35.1)	48 (64.9)	

χ^2^ Chi Square value, *df* degree of freedom, *p* significance, p^1^ based on difference between two proportions

Exposure to SHS at home - sex 5, age 5, ethnicity 11, presence of smokers 5, father’s employment status 15, mother’s employment status 16

Exposure to SHS in enclosed public places - age 2, ethnicity 6, presence of smokers 5, father’s employment status 10, mother’s employment status 11

Employment status of mother and presence of a smoker at home were the only variables that were significantly associated with higher odds of exposure to SHS at home in univariable regression. These associations remained significant once controlling for all other independent variables. Those whose mothers were unemployed had odds of being exposed to SHS at home 3.1 times those whose mothers were employed, and those who had a smoker in the home had 11.0 times the odds of being exposed to SHS at home, compared to those without a smoker in the household (Table 3).

**Table 3:** Odds ratios from logistic regression, with 95% confidence intervals, on the odds of exposure to second hand smoke at home for socioeconomic and individual factors amongst schoolchildren, Hanwella, Colombo, Sri Lanka

Factors	Model 1 OR Univariate	95% CI	Model 2 OR (+Age Sex Ethnicity)	95% CI	Model 3 OR (All factors)	95% CI
Sex						
Male (ref)	1		1		1	
Female	1.39	0.77–2.52	1.31	0.72–2.40	0.75	0.3–1.54
Age						
14 years (ref)	1		1		1	
15 years	1.05	0.58–1.89	1.21	0.66–2.23	1.43	0.71–2.87
Ethnicity						
Non- Sinhalese (ref)	1		1		1	
Sinhalese	1.52	0.72–3.21	1.54	0.72–3.29	1.14	0.48–2.69
Employment status of father						
Employed (ref)	1		1		1	
Unemployed	0.65	0.14–2.98	0.59	0.13–2.74	0.33	0.05–1.99
Employment status of mother						
Employed (ref)	1		1		1	
Unemployed	2.26**	1.21–4.22	2.32**	1.22–4.38	3.13***	1.51–6.47
Presence of smokers in household						
Not present (ref)	1		1		1	
Present	7.60****	3.84–15.03	8.25****	3.99–17.04	11.04****	4.97–24.52

Model 1: Univariable analysis

Model 2: Multivariable with age, sex and ethnicity (ORs presented for age, sex and ethnicity come from models including those variables only)

Model 3: Multivariable, including all variables

Significance level **p < 0.01, ***p < 0.005, ****p < 0.001,

*OR* odds ratio, *CI* Confidence Interval

Non-Sinhalese ethnicity and the presence of a smoker at home were significantly associated with higher odds of being exposed to SHS in enclosed public places in univariable regression. These were also significant in final models when controlling for all other factors, along with unemployment status of mother. Those of non-Sinhalese ethnicity had odds of being exposed to SHS in enclosed public places 2.3 times those of Sinhalese ethnicity. Individuals whose mothers were unemployed had odds of being exposed to SHS in public places twice those whose mothers were employed, and those who had a smoker in the home had odds 2.5 times the odds of being exposed to SHS in public than those who did not have a smoker at home (Table 4).

**Table 4:** Odds ratios from logistic regression, with 95% confidence intervals, on the odds of exposure to second hand smoke in public places for socioeconomic and individual factors amongst schoolchildren, Hanwella, Colombo, Sri Lanka

Factors	Model 1 OR Univariate	95% CI	Model 2 OR (+Age Sex Ethnicity)	95% CI	Model 3 OR (All factors)	95% CI
Sex						
Male (ref)	1		1		1	
Female	1.32	0.79–2.20	1.41	0.83–2.38	1.12	0.63–1.98
Age						
14 years (ref)	1		1		1	
15 years	1.31	0.78–2.19	1.21	0.71–2.05	1.38	0.78–2.41
Ethnicity						
Sinhalese (ref)	1		1		1	
Non-Sinhalese	1.88*	1.08–3.29	1.76	0.98–3.13	2.34**	1.26–4.36
Employment status of father						
Employed (ref)	1		1		1	
Unemployed	1.32	0.44–3.93	1.51	0.49–4.65	1.52	0.46–4.96
Employment status of mother						
Employed (ref)	1		1		1	
Unemployed	1.77	1.00–3.12	1.83*	1.02–3.29	2.01*	1.09–3.67
Presence of smokers in household						
Not present (ref)	1		1		1	
Present	2.57***	1.35–4.91	2.75***	1.39–5.42	2.52**	1.24–5.10

Model 1: Univariable analysis

Model 2: Multivariable with age, sex and ethnicity (ORs presented for age, sex and ethnicity come from models including those variables only)

Model 3: Multivariable, including all variables

Significance level *p < 0.05, **p < 0.01, ***p < 0.005

*OR* odds ratio, *CI* Confidence Interval

## Discussion

This study sought to assess the prevalence of SHS exposure among schoolchildren aged 14 and 15 years (grades 9 and 10) and its association with selected sociodemographic and health behavioural risk factors. The results revealed that 17.4% of 14-year-old and 18.0% of 15-year-old students were exposed to SHS at home. Almost 30% of 15 year olds and 23% of 14 year olds were exposed to SHS in enclosed public places. Presence of smokers in the household was the strongest determinant of exposure to SHS at home and in public places. The present study is unique in that there has been no recent studies on the prevalence of exposure to SHS among schoolchildren in Sri Lanka, nor its determinants, despite several recent anti-smoking activities in schools and communities.

The prevalence of exposure to SHS at home among schoolchildren in Sri Lanka, found within this study, was within the range of those from previous studies in the country of between 16.3 and 30% [18, 19]. Exposure to SHS in enclosed public places (25.7%) was lower compared with previous Sri Lankan studies, which have reported prevalence between 53 and 68% [6, 18]. These differences may be due to several reasons. Previous studies cited here were conducted several years ago and anti-smoking laws in Sri Lanka [20], introduced in 2006, included strict enforcement of smoking bans in public places, which may have contributed to a reduction. Although the antismoking law in Sri Lanka has provisions for a smoking ban in enclosed public places, it does not include households, which may explain a drop in SHS in public places but not homes. However, we must also acknowledge the sample differences between our studies and earlier ones. We sampled from one MOH region in Colombo, whereas other studies either sampled more widely from Colombo district [19], or nationally [18].

Based on our results, it is clear that effective enforcement of the antismoking law is imperative. It is evident that exposure to SHS at home and in public places in Sri Lanka remains high, despite continuous community and school based anti-smoking programmes. Similar to previous studies [9–12], the present study also reported that the presence of smokers at home was the strongest predictor of exposure to SHS both in the household and in public places. Results from this study highlight an urgent need for intervention to protect children from SHS at home.

One of the main strengths of the present study is the use of a large sample representative of the population in the MOH area. The prevalence estimates obtained are therefore likely to represent closely SHS exposure among schoolchildren in grades 9 and 10 in that MOH. The study also had a high response rate with few missing data, hence bias attributable to non-response among the population studied are expected to be low. By requesting the teacher to leave the classroom and assuring that the data will be anonymous and confidential, we have attempted to minimize external influences and underreporting.

The present study has a number of limitations. First, the exposure to SHS was ascertained by questioning whether anyone has smoked tobacco inside their household/enclosed public place in their presence within the 7 days prior to data collection. Reported exposure status was not confirmed using biomarkers such as urinary cotinine, which may provide a more accurate measure on individual exposure. Although self-reported data on exposure to SHS have been thought to be unreliable, most studies use this approach [29] and confidentiality and careful wording of the questions has been found to improve accuracy, when validated against biochemical markers. No previous studies on SHS conducted in Sri Lanka has used biochemical markers to improve the reliability of the data and this remains an area that warrants further investigation. A study on primary schoolchildren in Turkey found good agreement between biochemical (urinary cotinine) and self-reported measurements [30]. In the absence of objective measures of smoke exposure, which are impractical for large population studies, self-reported data collection would appear to be the most feasible approach for assessing exposure to SHS. Duration of exposure was also not measured, which may be a limiting factor in estimating the burden of SHS related ill health. The study was cross sectional in nature, limiting our ability to infer causality, meaning we report only associations throughout. In addition, as the study was limited to one MOH area in Sri Lanka and data collected from schoolchildren in just two grades (9 and 10) from nine schools there may be concerns over the external validity and generalizability of the study to the whole Sri Lankan population. In addition, as with many studies, residual confounding may be an issue. The study was designed to examine certain factors previously found to be linked to SHS exposure, but did not include measures on all these factors. For example, we did not collect data on peer smoking or personal (individual) smoking behaviour. The collection of these data are always difficult, due to the sensitive nature of the behaviour, especially as it is illegal for those under the age of 16 to buy cigarettes in Sri Lanka [20, 21]. Previous data collections on health behaviours, including the piloting of surveys used here, demonstrate that students are unwilling to report on their own smoking behaviour and that this can affect data collection for the study. We therefore elected not to collect it in this study, but recognise it as an important determinant that should be a focus going forward. There may also be other determinants that we did not collect data on, including those that previous studies have also not identified. Lastly, we focused on SHS within this study and not total ETS. Although it is desirable to consider all ETS exposure, the self-reporting of this may be challenging as individuals may not always be aware that they have been exposed to THS. In the future, more objective measures of ETS exposure may overcome this limitation.

Future studies should investigate the unidentified determinants further, perhaps through qualitative enquiry, as well as considering the impact of peer behaviour on exposure to SHS. The objective measurement of smoke inhalation may be beneficial in avoiding reporting bias in the self-reported collecting of these data. In addition, studies should consider within country variation and recognize that large differences in prevalence and determinants may occur between regions in the same country. Finally, the impact of policy and legislation implementation should be evaluated, preferably through mixed-method approaches.

## Conclusion

Despite lower prevalence figures in the exposure to SHS than in previous studies in Sri Lanka, the findings from this study suggest that exposure is still too high. These data strengthen calls for the implementation and enforcement of the current anti-smoking legislation along with additional public health initiatives to protect children from SHS. The study found that exposure to SHS was higher in public places than at home. Non-Sinhalese ethnicity, unemployment status of the mother and presence of smokers at home were significant determinants of exposure to SHS in public. Our data support the provision of education for schoolchildren on the health risks of SHS. Enforcement of the antismoking legislation is imperative. Large-scale, nationwide studies are also needed in order to re-assess the effectiveness of such interventions.

## Additional files

Additional file 1: Questionnaire. (DOC 49 kb)

Additional file 2: **Figure S1.** Study recruitment. (DOC 28 kb)

## Abbreviations

CI: Confidence interval
ETS: Environmental Tobacco Smoke
MOH: Medical Officer of Health
SHS: Second hand smoke
