# Supplementary material for: Prevalence and correlates of exposure to second hand smoke (SHS) among 14 to 15 year old schoolchildren in a medical officer of health area in Sri Lanka
Source: BMC Public Health. 2018 Nov 7;18:1240. doi: 10.1186/s12889-018-6148-4 (PMC6222988; doi:10.1186/s12889-018-6148-4)
Supplement: Supplementary file 1 — Questionnaire. (DOC 49 kb) [file 12889_2018_6148_MOESM1_ESM.doc]

**Prevalence and correlates of exposure to second hand smoke (SHS) among 14 to 15 year old schoolchildren in a Medical Officer of Health area in Sri Lanka**

**Instructions for the student**

- This is a questionnaire to assess your exposure to passive smoking due to tobacco.
- The confidentiality of your answers is guaranteed. Neither your teacher nor your parent can identify your answer
- Please don’t write your name
- You are free to withdraw from answering at any time.
- Read the question carefully before answering.
- Think your own way of reacting and choose your response according to it. It may be a wrong way of behaving, but you shouldn’t feel dishonoured.
- Mark your response honestly. Write an answer when a blank space is provided. Mark with (X) in the appropriate box.
- Unless asked never give more than one answer.

Would you like to answer this questionnaire?

Yes Start answering

No Please return the questionnaire

**Socio demographic questionnaire**

| 1 | I am a  1. Boy 2. Girl |
| --- | --- |
| 2 | My grade Grade 9 Grade 10 |
| 3 | My ethnic group    1. Sinhalese 4. Moor  2. Sri Lankan Tamil 5. Other  3. Indian Tamil 6. Reject |
| 4 | My parent’s occupation  Mother   1. Yes 2. No   If yes, please mention the occupation ----------------------  Father     1. Yes 2. No   If yes, please mention the occupation ---------------------- |
| 5 | Presence of smokers at home   1. Yes 2. No |

Exposure to passive smoking

| 1 | During the last 7 days, did anyone smoke tobacco (cigarette bidi / cigars) inside my home in my presence?   1. Yes 2. No |
| --- | --- |
| 2 | During the last 7 days, did anyone smoke in an enclosed area (Eg: inside a building, in an office, inside a vehicle, in another house, in a hotel –even in a teashop) when I was present?   1. Yes 2. No |

Thank you for answering the questionnaire!

Signature of the examiner …………………… Date of data collection ……..D……M……Y
