# Supplementary material for: Prevalence and correlates of exposure to second hand smoke (SHS) among 14 to 15 year old schoolchildren in a medical officer of health area in Sri Lanka
Source: BMC Public Health. 2018 Nov 7;18:1240. doi: 10.1186/s12889-018-6148-4 (PMC6222988; doi:10.1186/s12889-018-6148-4)
Supplement: Supplementary file 2 — Figure S1. Study recruitment. (DOC 28 kb) [file 12889_2018_6148_MOESM2_ESM.doc]

| Students invited to participate (n=354)  Excluded (n=37)  No parental consent (n = 19)  Students absent (n = 18)  Agreed to participate the study (n=317)  Excluded (n=6)  Included in analysis (n=311) |
| --- |
